# Sourdough-Based Biotechnologies for the Production of Gluten-Free Foods

**DOI:** 10.3390/foods5030065

**Published:** 2016-09-20

**Authors:** Luana Nionelli, Carlo Giuseppe Rizzello

**Affiliations:** Department of Soil, Plant and Food Science, University of Bari Aldo Moro, I-70126 Bari, Italy; luana.nionelli@uniba.it

**Keywords:** sourdough, lactic acid bacteria, gluten, gluten-free, baked goods

## Abstract

Sourdough fermentation, a traditional biotechnology for making leavened baked goods, was almost completely replaced by the use of baker’s yeast and chemical leavening agents in the last century. Recently, it has been rediscovered by the scientific community, consumers, and producers, thanks to several effects on organoleptic, technological, nutritional, and functional features of cereal-based products. Acidification, proteolysis, and activation of endogenous enzymes cause several changes during sourdough fermentation, carried out by lactic acid bacteria and yeasts, which positively affect the overall quality of the baked goods. In particular, the hydrolysis of native proteins of the cereal flours may improve the functional features of baked goods. The wheat flour processed with fungal proteases and selected lactic acid bacteria was demonstrated to be safe for coeliac patients. This review article focuses on the biotechnologies that use selected sourdough lactic acid bacteria to potentially counteract the adverse reactions to gluten, and the risk of gluten contamination.

## 1. The Sourdough Fermentation

The use of sourdough as a natural starter for leavening goods is considered one of the oldest biotechnological processes in food fermentation [1]. Sourdough is a mixture of flour (e.g., wheat, rye), water, and other ingredients (e.g., NaCl) that is fermented by naturally occurring lactic acid bacteria (LAB) and yeasts. These microorganisms originate mainly from flours and processing equipment, but the resulting composition of the sourdough microbiota is determined by endogenous (e.g., chemical and enzyme composition of the flour) and exogenous (e.g., temperature, redox potential, water content, and duration of the fermentation process) factors [2]. In mature sourdoughs, LAB dominate, occurring at concentrations of >10^8^ cfu/g, whereas the number of yeasts is commonly one/two logarithmic cycles lower [3]. Sourdough fermentation positively influences all aspects of baked goods’ quality such as texture, aroma, nutritional properties, and shelf life. Recently, sourdough has been successfully applied for the improvement of the quality of naturally gluten-free (GF) bread due to the complex metabolic activity of LAB. Moreover, novel biotechnologies based on sourdough fermentation have been proposed for the complete degradation of gluten in cereal flours, rendering them suitable for the production of innovative GF products. Both these aspects, which are rapidly evolving thanks to the scientific community and the food industry, are taken into account and described in this review.

## 2. The Sourdough Lactic Acid Bacteria

Microbiological studies have revealed that more than 50 species of LAB and more than 25 species of yeasts, especially belonging to the genera *Saccharomyces* and *Candida*, occur in mature sourdoughs. Sourdough is considered a unique food ecosystem in that it (i) selects for LAB strains that are adapted to its environment and (ii) harbours LAB communities specific to each sourdough [4,5,6,7]. Representative genera of sourdough LAB are *Lactobacillus*, *Leuconostoc*, *Pediococcus*, and *Weissella* [8]. The largest biodiversity was found within the genus *Lactobacillus* and a relatively high number of species was discovered recently [2,4,9,10,11,12,13,14]. Depending on the protocols used for sourdough propagation, various microbial consortia of mainly obligate and facultative hetero-fermentative LAB are found. *Lactobacillus brevis*, *Lactobacillus fermentum*, *Lactobacillus paralimentarius*, *Lactobacillus plantarum*, *Lactobacillus pontis*, and, especially, *Lactobacillus sanfranciscensis*, considered to be a key sourdough bacterium [14], are commonly isolated from traditional sourdoughs.

## 3. Sourdough Properties and Functions

Beyond its natural and additive-free image, it is generally accepted that sourdough has various positive effects when used for manufacturing baked goods. Compared to other leavening agents (e.g., baker’s yeast), it improves the texture, flavour, nutritional value, and shelf life of bread [15]. Notwithstanding the role of sourdough yeasts, the main metabolic properties of LAB determining the above effects are briefly described.

### 3.1. Texture and Structure

Depending on the level of lactic acidification, sourdough fermentation leads to an increase in bread extensibility, softness, and volume [16,17,18,19,20]. Overall, sourdough fermentation improves the gas retention in bread dough [2,18]. Acidification affects the solubility of structure-forming components like gluten, starch, and arabinoxylans, and positively interferes with the activity of endogenous enzymes [21]. Acidification affects the mixing behaviour of the dough: at low pH, a shorter mixing time and less stability than normal dough are achieved [21].

### 3.2. Flavour

The fermentation of soluble carbohydrates (e.g., maltose, glucose, and fructose), metabolism of nitrogenous compounds, and generation of volatile compounds by sourdough LAB directly or indirectly influence the flavour of baked goods. Beyond the Embden–Meyerhof–Parnas (EMP, facultative hetero-fermentative strains) and phosphogluconate (obligate hetero-fermentative strains) pathways, the use of external acceptors of electrons [22,23,24] or alternative energy sources [25], and the interactions with endogenous and exogenous enzymes [26] lead to different quotients of fermentation (molar ratio between lactic and acetic acids) that differently affect the flavour of baked goods. Overall, sourdough fermentation results in a large increase of free amino acids (FAAs), compared to the baker’s yeast process [7]. Proteolysis during sourdough fermentation includes the hydrolysis of proteins to intermediate-sized polypeptides and subsequent release of FAAs from polypeptides allowed by the LAB peptidase system [5,27]. Once liberated, FAAs contribute directly to flavour or are further subjected to chemical conversion during baking or enzymatic catabolism [28], thus leading to the synthesis of flavour volatile compounds. Within the catabolism of FAAs, the expression of the arginine deiminase (ADI) pathway in sourdough LAB [29] enhances the growth and tolerance to acid stress, and, especially, increases the synthesis of ornithine, which is the precursor of the 2-acetyl-pyrroline, responsible for the roasted note of the wheat bread crust [5].

Among the compounds having a key role in baked goods’ flavour formation, homo-fermentative LAB mainly synthesize diacetyl, acetaldehyde, and hexanal, while hetero-fermentative strains mainly produce ethyl acetate, alcohols, and aldehydes. Iso-alcohols with their respective aldehydes and ethyl-acetate are characteristic volatile compounds of yeast fermentation [30,31].

### 3.3. Nutrition

Sourdough fermentation modifies nutritional features of cereals by (i) improving texture and palatability of wholegrain and fibre-rich bread; (ii) stabilizing or increasing levels of bioactive compounds; (iii) decreasing starch bioavailability (low glycaemic index products); and (iv) improving mineral bioavailability [32]. Lactic acidification increases the levels of bioactive compounds (e.g., phenolic compounds) [33] and causes the degradation of phytate, increasing mineral bioavailability [34,35]. Furthermore, lactic acidification also increases the magnesium and phosphorus solubility [32] and has been found to be a protective factor for β-glucan in bread. Organic acids such as those produced during sourdough fermentation have also been shown to play a role in the postprandial glycaemic responses. The presence of lactic acid during heat treatment promotes interactions between starch and gluten, reducing starch bioavailability and, consequently, the glycaemic index of baked goods [36,37].

### 3.4. Shelf Life

The improvement of the loaf’s specific volume and crumb softness by sourdough fermentation have been associated with the decrease of the rate of bread going stale [15,16]. Starch molecules may be affected by enzymes synthesized by LAB, causing a variation in the retrogradation properties of the starch and decreasing the rate of going stale. Besides going stale, microbial spoilage by bacteria, and especially moulds, remain responsible for huge economic losses in the bakery industries. Acidification through sourdough fermentation has been shown to inhibit the endospore germination and growth of *Bacillus* sp. responsible for rope spoilage [38]. Besides various compounds (e.g., organic acids, hydrogen peroxide, diacetyl), sourdough LAB may inhibit the growth of other related micro-organisms by synthesizing bacteriocins, bacteriocin-like inhibitory substances (BLIS) [5,39], and low-molecular mass antibiotics such as the reutericyclin of *L. reuteri* LTH2584 [40]. A number of antifungal metabolites, e.g., a mixture of organic acids (acetic, caproic, and formic acids), cyclic dipeptides, phenyllactic acid, proteinaceous compounds, and 3-hydroxylated fatty acids, are potentially synthesized by LAB [41,42,43,44,45] acting against moulds responsible for bread spoilage. Different peptides with antifungal activity were identified in the water-soluble extracts of wheat flour fermented with LAB, as the results of the proteolytic activity on the native wheat proteins show [45,46]. Overall, all the peptides produced by LAB in wheat-based matrices were characterized by a large inhibitory spectrum against species that commonly contaminate baked goods and bakeries [45,46,47], allowed a long storage of bread (at least 21–28 days).

### 3.5. Functional Properties

During sourdough fermentation, LAB may also produce bioactive compounds such as peptides and amino acid derivatives (e.g., γ-amino butyric acid) with various functionalities, and potentially prebiotic exo-polysaccharides.

The potential of sourdough lactic acid bacteria to release lunasin, a strong anti-tumoural peptide, during fermentation of cereal flours, was recently exploited [48]. Recently, flours obtained from different legume species were subjected to fermentation with selected LAB strains, showing the release of lunasin-like polypeptides, as the consequence of the proteolysis of native proteins [49]. A marked inhibitory effect on the proliferation of human adenocarcinoma Caco-2 cells was observed using extracts from fermented legume doughs (up to 70%) [49].

The capacity of selected lactic acid bacteria to release antioxidant peptides was shown during the fermentation of various cereal flours. Purified peptides showed ex vivo antioxidant activity on mouse fibroblasts artificially subjected to oxidative stress [50].

Lactic acid bacteria selected for proteolytic activity were used for wheat and rye fermentation with the aim of producing anti-hypertensive peptides. A strong ACE-inhibitory activity was found when fermenting flours under semi-liquid conditions and, especially, when using whole wheat flour [51].

## 4. Adverse Reactions to Gluten

Coeliac disease (CD), also known as coeliac sprue and gluten-sensitive enteropathy, is a food hypersensitivity disorder caused by an inflammatory response to wheat gluten and similar proteins of barley and rye [52]. In recent years, the view of CD has undergone a profound revision. Nowadays, CD is considered more than a just a gluten-sensitive enteropathy but a systemic immune-mediated disorder elicited by gluten and related prolamines in genetically susceptible individuals. The common denominator for all subjects with CD is the presence of a variable combination of gluten-dependent clinical manifestations, specific autoantibodies (anti-tissue transglutaminase [TG]2, anti-endomysium [EMA] antibodies), HLA-DQ2, and/or DQ8 haplotypes, and different degrees of enteropathy, ranging from lymphocytic infiltration of the epithelium to complete villous atrophy [53]. Reports of CD date back to the first century AD [54], but it was not until 1888 that Samuel Gee gave the classical description of the disease [55], and it was only in the 1930s that Willem-Karel Dicke observed that removal of wheat from the diet alleviated the symptoms and signs of CD [56]. Nowadays, the prevalence of CD worldwide is increasing; it is estimated to be 0.5%–2.0% in most of the European countries and the United States. Such a rate establishes CD as one of the most common food intolerances [57]. Gluten may also induce other pathological conditions, such as wheat allergy (WA) [58], which is an immunoglobulin (Ig)E-mediated disease also well characterized from the immunological and clinical point of view but completely unrelated to CD. More recently, attention was given to another entity, gluten sensitivity (GS), for which the limits and possible overlap with CD are still poorly defined [59]. GS subjects are unable to tolerate gluten and develop an adverse reaction when eating gluten that usually, and differently from CD, does not lead to damage in the small intestine. A number of morphological, functional, and immunological disorders have been considered under the definition of GS that miss one or more of the key CD criteria (enteropathy, associated HLA haplotypes, and presence of anti-TG2 antibodies), but respond to gluten exclusion. Nowadays, the only effective treatment for CD consists of a lifelong gluten-free diet (GFD). The regression of symptoms in response to the GFD was also shown in WA and GS subjects. Nevertheless, gluten is a common, and in many countries unlabelled, ingredient in the human diet, presenting a big challenge for CD and WA patients, and GS subjects; therefore, there is an increasing need to develop safe and effective alternatives. Beyond genetic predisposition, several environmental factors influence adverse reactions to gluten. Recent epidemiological studies show that the introduction of gluten-containing grains, which occurred about 10,000 years ago with the advent of agriculture, represented an evolutionary challenge that created the conditions for human diseases related to gluten exposure [60]. More recently, cereal food technology has changed dramatically by influencing the daily diet of entire populations previously not exposed to high concentrations of gluten. Cereal baked goods are currently manufactured by a very accelerated process where long fermentations by sourdough, a cocktail of acidifying and proteolytic lactic acid bacteria with or without *Saccharomyces cerevisiae*, were almost totally replaced by the indiscriminate use of chemical and/or baker’s yeast leavening agents. Under these technological circumstances, cereal components (e.g., proteins) are subjected to very mild or absent degradation during manufacture, resulting, probably, in less digestible foods compared to traditional and ancient sourdough baked goods [7].

## 5. Applications of Sourdough in Gluten-Free Products

Gluten is one of the most important structure-building protein complexes responsible for the quality and structure of wheat-based products. Its viscoelastic properties render the development of GF dough having similar quality and structural properties a highly challenging task, and form a major industrial hurdle [61]. The development of GF products to date remains a technologically intriguing area for researchers as well as the food industry [61]. Indeed, dough produced from GF formulations lacks a cohesive and elastic nature due to the absence of gluten, which makes industrial handling of dough a greater challenge. However, in the recent past, various alternative approaches (e.g., high-pressure, extrusion, enzymatic treatments) have been adopted to modify functional attributes necessary for GF dough development. These approaches could provide potential solutions in the development of GF products for the coeliac community globally [61].

The sourdough biotechnology has been applied to the manufacture of gluten-free products with the aim of improving their sensory and nutritional features. Overall, the gluten-free products available on the market are of low quality, exhibiting poor mouth-feel and flavour [62].

Since they do not contain gluten, and are mainly starch-based, GF bread products go stale more rapidly than gluten-containing bread [63]. In addition, when limiting the use of gluten-free flours to the most common sources (e.g., rice, corn, and starches), nutrient deficiencies may occur due to the very low dietary fibre content and excess calories [64]. Nevertheless, the current literature indicates a limited number of papers dealing with the use of sourdough in gluten-free goods. The few available results indicate that sourdough has a positive effect on the baking quality, particularly on volume, texture, and flavour. The influence of sourdoughs fermented by different LAB strains on the textural quality of gluten-free bread was evaluated during storage and compared to that of chemically acidified or non-acidified doughs [17,18]. The growth of selected LAB in gluten-free batters was similar to that reported for wheat sourdoughs [18]. Sourdough fermentation caused an increase in the dough elasticity and delayed the process of going stale [65]. These effects were mainly attributed to the breakdown of non-gluten proteins and starch components by sourdough LAB. Based on triangle tests, gluten-free sourdough bread was discriminated from the control bread and clearly preferred. In a recent patent [66] *L. sanfranciscensis* LS40 and LS41, and *L. plantarum* CF1, previously isolated from traditional sourdoughs, were selected. This microbial mixture was used to ferment gluten-free ingredients (e.g., corn starch, rice, buckwheat, and millet flours) and compared to baker’s yeast fermentation. The sourdough fermentation allowed us to: (i) completely degrade about 300 ppm of gluten, eventually present as contaminant; (ii) increase by about 10-fold the concentration of FAAs; (iii) increase by about 10-fold the phytase activity during fermentation; and (iv) improve the sensory characteristics of the resulting bread as evaluated by descriptive analysis. 

A Type-I GF sourdough was obtained using only naturally GF flours, through the typical backslopping procedure [67]. After few refreshments in controlled conditions, the sourdough presented a stable association between *L. sanfranciscensis* and *Candida humilis*, constant fermentation times, and technological properties (in terms of dough consistency, dough maximum height, CO_2_ production, and retention) [67]. The results showed that a traditional sourdough biotechnology can also be used to improve the overall quality of GF baked products [67].

The positive contribution of sourdough could be exploited for the design of high-quality GF bread from various GF cereals and pseudocereals [68]. For example, the effect of adding fresh and freeze-dried amaranth and buckwheat into the GF bread formula has recently been investigated [68,69], showing several advantages in taste and aroma [68]. Sourdoughs obtained with teff and buckwheat (through the use of a selected *L. helveticus* strain) were used for the making of experimental GF breads that were characterized by sensory analysis and sensory tesst showing enhanced bread aroma and increased fruity, cereal, and toasty notes [68]. Chestnut flour was subjected to a spontaneous fermentation and a typical backslopping procedure [70], obtaining a sourdough that was included in the formulation of corn-based GF bread, along with a volume increase, a decrease of the crust hardness, and a longer shelf life [70]. A *Lactobacillus amylovorus* strain was employed as a starter culture for gluten-free quinoa sourdough bread under pilot-plant conditions to extend the microbial shelf life [71]. It was demonstrated that the use of quinoa sourdough extended the mould-free shelf-life up to four days compared to the non-acidified control, thanks to the high concentration of 4-hydroxyphenyllactic acid, phloretic acid, 3-phenyllactic acid, and hydroferulic acid [71]. Evaluation of bread characteristics such as specific volume or crumb hardness revealed that the addition of *L. amylovorus*-fermented sourdough also improved bread quality [71].

Breads based on gluten-free buckwheat, quinoa, sorghum, and teff flours were produced with the addition of sourdough fermented with exopolysaccharide (EPS) producing *Weissella cibaria* MG1, showing that the acidification increased crumb porosity and decreased hardness [72]. Moreover, the authors reported that the staling rate was significantly reduced [72]. In any case, the use of sourdough decreased the degree of in vitro starch hydrolysis (and, consequently, the predicted glycaemic index) [73].

The increasing demand for high-quality gluten-free baked goods, clean labels, and natural products points to the need for new approaches in GF bread making [74]. Overall, the positive effects of sourdough, extensively studied for traditional baking, overlap those found using sourdough in GF baking. The microbiological and qualitative characterization suggests that the metabolic activities of the sourdough microbiota are still retained during fermentation of GF matrices [74]. Thus, due to the sensory, texture, and nutritional improvements, the large-scale industrial use of sourdough for the manufacture of gluten-free goods can be recommended.

## 6. Sourdough Lactic Acid Bacteria as a Tool for Detoxifying Gluten in Wheat-Based Foods

Beyond genetic predisposition, several environmental factors influence CD prevalence. Recent epidemiological studies show that, besides being frequently found in countries where individuals are mostly of European origin, CD is a common disorder in many areas of the developing world (the Middle East, North Africa, South and East Asia, and Latin America). As mentioned before, the modern food industry has replaced sourdough biotechnology with the large-scale use of chemical and/or baker’s yeast leavening agents [7]. Nevertheless, the traditional biotechnology of sourdough bread making has been recently exploited for the capacity to degrade toxic epitopes during food processing. This “food technological approach,” together with other methods aiming at the hydrolysis of toxic gluten peptides prior to ingestion, has been proposed and developed as an alternative to the hydrolysis of gluten peptides after ingestion in the gastrointestinal tract (the “medical approach”) [75]. Extensive research in this field is ongoing at the authors’ laboratory in a joint project with medicine specialists to show the potential of proteolytic enzymes of sourdough LAB as it has been widely shown for prolyl endopeptidases (PePs) of *Flavobacterium meningosepticum* [76], *Myxococcus xanthus* [77], and *Aspergillus niger* [78,79].

### 6.1. Use of Selected Lactic Acid Bacteria for Gluten Degradation

Since the last decade, several studies [80,81,82,83] have been carried out aiming at showing the capacity of proteolytic enzymes, mainly peptidases, of selected sourdough lactobacilli to degrade gluten during food processing. The use of pooled cell suspensions and cell-free extracts obtained by different lactic acid bacteria strains was investigated [84]. Although a number of in vitro (e.g., agglutination and Caco-2/TC assays), ex vivo (biopsy-derived T cells), and acute in vivo (intestinal permeability) tests were carried out, the above results [80,81,82,83] only showed a marked decrease of the gliadin fraction, but not a complete degradation. Recently, it was shown that a traditional sourdough fermentation, leading to a partial gluten hydrolysis of wheat flour proteins, is not able to prevent the interaction of transglutaminase 2 with α2-gliadin or gluten; thus it cannot be considered safe for making GF products [85]. This route might be helpful to eliminate the risk of cross-contamination of gluten-free products but not to completely eliminate the toxicity of wheat flour.

Nevertheless, further efforts were made to increase the hydrolysing capacity of sourdough LAB. Together with two fungal proteases (obtained from *Aspergillus niger* and *A. oryzae*), routinely used in bread making, other lactobacilli strains, characterized by a marked peptidase activity towards Pro-rich peptides [86], were used during long-time fermentation of semi-liquid wheat flour doughs. As determined by R5-sandwich and competitive ELISA, the residual concentration of gluten in the fermented sourdough was <20 ppm, as required by the standard of the Codex Alimentarius Commission for gluten-free products. Nevertheless, due to the limitation of the R5-ELISA methods, further investigations based on in vitro, ex vivo, and in vivo assays were performed in order to assess the complete degradation of all the protein epitopes involved in the pathology, including those of glutenins. Two-dimensional electrophoresis and MALDI-TOF mass spectrometry analyses showed the complete hydrolysis of albumins/globulins and gliadins [87]. After hydrolysis, the spray-dried flour from fermented sourdough was mainly a mixture of water/salt-soluble low molecular weight peptides and amino acids. Many chemical analyses and ex vivo tests on human cell cultures confirmed the complete detoxification of gluten [87,88]. After sourdough fermentation, the water was removed and the pre-treated wheat flour was used for bread making by using baker’s yeast and structuring agents (e.g., gums). Structuring ingredients are necessary since the resulting gluten network was completely disrupted [89]. This sourdough bread was compared to baker’s yeast bread made with non-treated flour and without structuring agents. The specific loaf volume of sourdough bread was similar to that of baker’s yeast bread and showed the typical flavour of the sourdough wheat bread, as judged by an internal panel test [87,88]. Recently, a bread made with semolina rendered GF by the protocol here described was compared to commercial GF breads made with naturally GF ingredients [90]. Beyond the huge potential of market expansion, the main advantages of wheat flour rendered GF are the high availability of FAAs, the high protein digestibility, the low starch hydrolysis index, and the better technological properties of bread compared to the commercial GF products currently on the market [90]. Vitamins, minerals, and dietary fibre profiles are comparable to those of gluten-containing wheat bread. The sensory profile, determined by a panel test, can be considered the most similar to those of conventional baked goods, showing all the classic attributes of sourdough bread [90].

### 6.2. Mechanisms of Detoxification and in Vivo Tests

**Figure 1:**
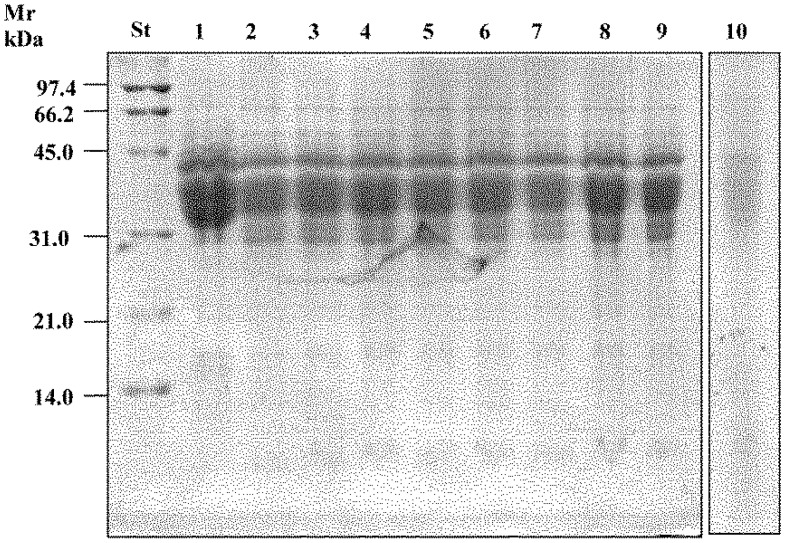
SDS-PAGE analysis of gliadins polypeptides from wheat flour doughs incubated for 24 h with the different cell preparations (10^9^ cfu/mL) that composed the VSL#3 preparation. Protein standard (St). Chemically acidified dough (1); doughs incubated with cells of *Bifidobacterium longum* (2); *Lactobacillus delbrueckii* subsp. *bulgaricus* (3); *L. plantarum* (4); *L. casei* (5); *B. infantis* (6); *L. acidophilus* (7); *Streptococcus thermophilus* (8); *B. breve* (9); and VSL#3 preparation (10). Adapted from De Angelis et al. [96].

Activity of fungal proteases was responsible for the primary proteolysis, liberating various sizes of polypeptides. The large proportion of proline residues in the amino acid sequences characterizing the toxic peptides make them extremely resistant to further hydrolysis [91,92,93]. The specific cyclic structure of the proline imposes many restrictions on the structural aspects of peptides. To adequately deal with such peptides, a group of specific peptidases is necessary to hydrolyse peptide bonds. Prolyl endopeptidases (PEPs) of microbial origin are endoproteolytic enzymes, which, in contrast to human gastrointestinal proteases, may readily cleave Pro-rich immune-stimulatory gluten peptides [93]. Through a complex system of ABC and ATP transporters, gluten peptides are moved across the cytoplasmic membrane of sourdough lactobacilli. Already a few minutes after entry, the concentration of polypeptides markedly decreases, to about 100 times lower than that of the environment [94]. A pool of intracellular peptidases of the selected sourdough lactobacilli was used to simulate hydrolysis towards the 33-mer epitope. The combined activity of peptidases was responsible for the complete degradation of the 33-mer or other synthetic immunogenic peptides, which occurred within 14 h of incubation [94]. Lactic acid bacteria possess a very complex peptidase system [95], although not a unique strain that may possess the entire pattern of peptidases needed for hydrolysing all the potential peptides where Pro is involved. Nevertheless, the hydrolysing capacity was lost when individual strains were tested, confirming that no single strain contains the entire portfolio of peptidases necessary to degrade Pro-rich polypeptides (Figure 1). Sweet baked goods were made using the complete hydrolysed wheat flour. Two clinical challenges were carried out on coeliac patients, who ingested the equivalent of about 8 or 10 g of native gluten per day for 60 days [96,97]. Haematology, serology, and intestinal permeability analyses showed complete tolerance by all coeliac patients during the whole time. None of the CD patients had clinical complaints and none produced anti-TG2 antibodies or had modification of the small intestinal mucosa compared to the pre-challenge situation [96]. No increase of CD3 and gamma delta cells was found, and the Marsh grade was unchanged after the challenge.

### 6.3. Use of Detoxified Wheat Flour for Pasta Making

**Figure 2:**
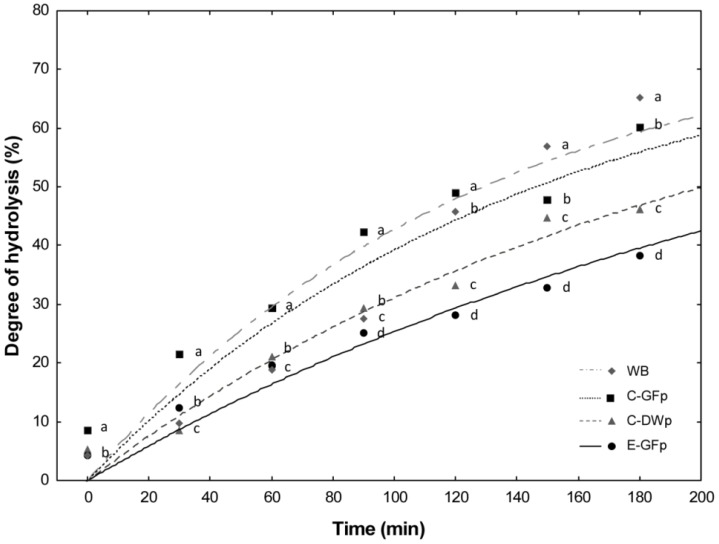
Rate of starch hydrolysis of pasta following chewing, incubation with pepsin, and further incubation with pancreatic α-amilase in dialysis tubing. E-GFp: experimental gluten-free pasta; C-GFp: commercial gluten-free pasta; C-DWp: commercial durum wheat pasta; WB: white wheat bread (reference). ^a–d^ Values obtained at the same time with different superscript letters differ significantly (*p < 0.05*). Adapted from Curiel et al. [93].

Wheat flour, which was rendered gluten-free by sourdough LAB fermentation and fungal proteases, was used for manufacturing experimental gluten-free pasta (E-GFp), according to a traditional process with a low-temperature drying cycle [98]. Chemical, technology, structural, nutritional, and sensory features were characterized and compared with those of commercial gluten-free (C-GFp) and durum wheat pasta (C-DWp). E-GFp showed rapid water uptake and shorter optimal cooking time compared to the other pastas. Despite the absence of the gluten network, the supplementation with pre-gelatinized rice flour allowed structural properties of E-GFp, which were comparable to those of C-GFp [98]. The in vitro protein digestibility of E-GFp had the best results. Probably due to proteolysis during sourdough fermentation, the chemical scores, essential amino acids profile, biological value, and nutritional index of E-GFp were higher than those of C-DWp. The hydrolysis index (HI) of E-GFp was about 30% lower than that found for C-GFp (Figure 2). As shown by sensory analysis, the characteristics of E-GFp were acceptable. This novel pasta has rather good structural and sensory properties, enhanced digestibility, low HI, and high nutritional quality [98]. The manufacture of E-GFp should be promising to expand the choice of gluten-free foods, which combine sensory and nutritional properties [98].

## 7. Conclusions

Compliance with a gluten-free diet is an extremely challenging task, given a number of problems related to cross-contamination, lack of clear food labelling policies, and poor quality of gluten-free products compared to their gluten-rich counterpart. Even if the exploitation of sourdough in gluten-free systems is still in its infancy, available data indicate that sourdough may be considered a technological tool for improving the texture and flavour of gluten-free products. Indeed, it was demonstrated that the application of sourdough biotechnology to naturally GF ingredients, including GF cereals, pseudocereals, and legumes, may improve the sensory, technological, nutritional, and functional features of final products, similarly to the positive effects described for the gluten-containing matrices.

Besides the application to naturally GF flours, the setup of alternative biotechnologies based on sourdough fermentation is an active area of research that may provide novel possibilities to GF product development in the near future [61,94]. Indeed, the use of sourdough LAB was first proposed with the aim of eliminating traces of gluten epitopes in processed foods and will minimize the long-term risks of a multitude of individuals affected by CD worldwide. Recently, a novel biotechnology process to completely hydrolyse gluten in wheat flour [87] was optimized, patented [99], and industrialized, and GF baked goods made with rendered GF wheat flour are now available on the global market, providing new options for the food industry and consumers [88,90].

Together with recent medical and technological advances, sourdough-based biotechnology could contribute to improve the quality of life of coeliac patients in the near future.
